# Factors affecting the use of dental services among Arab children in Israel: a qualitative study

**DOI:** 10.1186/s13584-023-00579-4

**Published:** 2023-09-04

**Authors:** Mohammad Khatib, Yael Ashkenazi, Yoav Loeff, Shlomo Paul Zusman, Lena Natapov

**Affiliations:** 1 Zefat Academic College, Jerusalem St. 11, P.O Box 160, 13206 Zefat, Israel; 2 Myers-JDC-Brookdale Institute, Jerusalem, Israel; 3 Division of Dental Health, Ministry of Health, Jerusalem, Israel

**Keywords:** Dental care, Use of services, Arab children

## Abstract

**Background:**

In 2010, Israel reformed its hitherto dominantly privately financed dental services and included preventative and restorative dental care for children in the publicly-funded basket of healthcare services. A survey conducted by Brookdale Institute, found that only 67% of low-income Israeli-Arab children were using the new service (compared to 85% of Jewish children) while the majority of others continue using privately funded services. The aim of this study is to explore and explain Israeli-Arab children’s low utilization of publicly-funded preventive and restorative dental care.

**Methods:**

A qualitative study designed to describe and understand the parents’ motivations and choices. As a preliminary stage, eight semi-structured interviews were conducted with directors of HMO dental departments and Israeli-Arab dentists. In the second stage, ten one-on-one interviews with parents, and five focus group discussions with 55 parents held on February–March 2017. All discussions in the focus groups were conducted in Arabic and each group was moderated by one of the research team accompanied by another person who documented and recorded the discussion. All interviews and discussions were recorded, transcribed in full and translated into Hebrew.

**Results:**

The prevalent attitude is that one goes to the dentist only when there is a serious problem. The importance of preventive care is not appreciated. A childhood fear of the drill is very remembered and passed onto the children. Social and cultural factors such as kinship with service providers (GPs and dentists) influence the choice and utilization of health services. Economic barriers are still existing since even the small co-payment is daunting to low-income parents of large families. Provision of the public service is inadequate in some Arab villages.

**Conclusions:**

The extension of Israel's National Health Insurance Law’s basket of services to include dental care for children, while important, is not sufficiently embraced by Israeli Arab children. The remaining barriers include poor living conditions, low educational level that requires very clear sharing of information about the new service, and the resistance of cultural and social traditions. Public dental services providers should focus on conveying relevant oral health messages to the parents too, either through their children or directly.

## Background

In 2004 the World Health Organization (WHO) set global targets for 2020 for the promotion of oral and dental health. Various countries have adjusted their national oral health policy accordingly, focusing on reducing tooth decay rates, increasing early diagnosis and detection, improving oral and dental health, and more.

Israel has already started taking steps in this direction in July 2010, when preventative and restorative dental treatments for children were included in the publicly-funded basket of healthcare services of the National Health Insurance Law (NHIL), which are provided free or at a small co-payment by four Health Management Organizations (HMOs) to all Israeli residents. This reform was motivated by a concern that, in the privately financed system, many families were foregoing necessary dental services due to cost barriers, and that these barriers were especially problematic for low-income families [1].

The services were initially added to the basket for children up to the age of 8 and gradually extended, reaching 18 years-of-age in 2019. The basket includes preventive services (e.g. periodic check-ups, consultation and treatment planning, X-rays, scaling and root planning, pits and fissure sealing, and fluoride treatment) with no co-payment at all and low (about 6 US$) co-payment restorative services (e.g. emergency treatment, restorations, pulp treatment including root canal, extractions, nitrous sedation). The HMOs are responsible for providing the services either in their own clinics or by contracted private dentists.

Most of the dental services in Arab towns and villages are provided by contracted dentists, yet HMO's contracted clinics may not be available in every small town or village [2], which may affect accessibility among the lower socio-economic strata.

Since 2010, the School Dental Health Services provided by local authorities and funded by the Ministry of Health (MoH), have been significantly expanded. This service operates in kindergartens and schools and includes an annual screening check-up, followed by notification to parents about their children's oral health status and referral to dental clinic when needed, dental health education in the classrooms, including tooth brushing instructions and toothbrushes and a toothpaste distribution.

Three years after the reform, the Myers-JDC-Brookdale Institute, in collaboration with the Ministry of Health, conducted a survey to learn to what extent children in Israel were using the publicly-financed services, and what were the barriers to utilization among different population groups [3]. The survey found high uptake of the new service: 70% of children aged 2–11 who visited a dentist used the new publicly-financed service. However, whereas 85% of low-income Jewish children were using the new service, only 67% of Israeli-Arab children from the same income stratum were doing so, while the majority of others continued using privately funded services. In addition, only 23% of low-income Israel-Arab children went to routine check-ups, compared to 44% of low-income Jewish children. Earlier epidemiological surveys (1989, 2002 and 2007) found that Israeli-Arab children had more tooth decay, more damaged and missing teeth, and fewer filled teeth than Jewish children—possibly because of fewer visits to a dentist [2, 4]. A 2014–2015 survey of 1700 five-year-old children conducted after the dental care reform found a mean DMFT (Decayed, Missing and filled Teeth) caries measure index among Jewish children of 2.21 compared to 4.38 among Israeli-Arab children, while the majority of whose caries were untreated. Thus, the dental health disparity between Jewish and Israeli-Arab children continued to be substantial [5].

In 2017, Israeli-Arabs comprised 21% of Israel’s population [6] and the great majority lived in Israeli-Arab towns and villages. Most are of low socioeconomic status [7] and have, relative to Jewish Israelis, high levels of unemployment and poverty and low levels of social service provision [8, 9]. The large majority of households are in the three lowest income deciles, with a correspondingly low level of schooling [10].

Numerous studies have demonstrated a health status disparity between Israel’s Jewish and Arab citizens, which shows itself in the latter’s higher mortality and morbidity rates and lower life expectancy [11, 12]. There are also significant differences between the two population groups in their health behaviours—smoking, physical activity, consumption of fruit and vegetables [13] and their use of healthcare services [14, 15].

The present study was designed to explore and explain Israeli-Arab children’s low utilisation of preventive dental care and relatively low up take of publicly-financed dental care. Specifically, the study sought to—

- Understand how Israeli-Arabs parents perceive the importance of maintaining dental health and the steps that need to be taken for this purpose;
- Understand why the parents rarely take their children for dental check-ups;
- Understand the factors and judgements influencing their choice of a dental health service for their children;
- Identify obstacles to publicly-financed dental care utilization among this group

## Methods

The aim of this study was to describe and understand a complex reality from the point of view of different stakeholders. To do this, the qualitative method was used, which was based on semi-structured in-depth interviews and discussions in focus groups.

73 respondents participated in the study, which was conducted in three stages:

In the first stage (a preliminary stage), 8 semi-structured in-depth interviews were conducted with directors of HMO dental departments and Israeli-Arab dentists, both HMO employees and self-employed. For this purpose, an interview guide—for all interviewees—was prepared that included 10 questions about various issues such as dental services provided by the HMO to the Arab population, availability of and access to dental services in Israeli-Arab communities, their take-up by these communities’ residents, difficulties, barriers, and challenges in the provision of dental services, the use of preventive dental medicine by the Arab public.

The second stage of the study was in-depth face-to-face interviews with 10 parents (5 mothers and 5 fathers) from different Arab localities. The interviews were conducted between November 2016 and January 2017 and each interview lasted 20–30 min. Eight of the interviews were conducted in Arabic and two in Hebrew.

The main topics included in the interviews with the parents, both in the individual interviews and in the focus groups, were the way in which decisions are made in the family regarding the care of the children's teeth, experiences in receiving treatment, perceptions about what parents can do to maintain their children’s dental health, promoting factors and barriers to maintaining dental health and use of public dental health services, the relationship between dental health and general health and the way to choose a dentist and treatment clinic.

The third stage included 5 focus groups in which 55 participants from different Arab localities, all mothers—except for two fathers—who were recruited by appeals to various community institutions. Each focus group included 7–16 participants and all the discussions were conducted in February and March 2017 in Arabic and led by a member of the research team, with a second team member on hand to take notes and record the discussion. At the beginning of each interview and discussion, participants completed anonymously a brief questionnaire on their personal characteristics (age, gender, education, employment, number of children, and whether they had taken their children to a dentist and received treatment for them during the year before the discussion). All interviews and the focus group discussions were recorded, translated, and transcribed in Hebrew.

A *content analysis* of the interviews and discussions was made in order to identify recurrent main themes, sort the material into categories, and identify tendencies and key concepts. Some of the categories of this analysis were predetermined by the face-to-face interview structure; others emerged from the content analysis itself.

The study was approved by the Brookdale Institute ethics committee.

## Results

- The preliminary stage

The aim of the interviews conducted with directors of HMO dental departments and Israeli-Arab dentists was to learn about the preventive and therapeutic dental services provided by the HMO to the Arab population, availability of and access to those services, patterns of use, difficulties, barriers, and challenges in the provision of dental services, from the directors and dentists point of view. The content analysis revealed four themes:

*The pattern of uptake:*

Most of the directors and dentists indicated that although the uptake of dental services by Jewish children is still higher than by Israeli-Arab children, the gap is narrowing. Israeli-Arab children are generally not brought for regular check-ups so they arrive at HMO clinics, overall, in a much worse state of dental health, when there is already a lot of decay and pain. As a result, the out-of-pocket cost to the parents is higher. This delay in seeking treatment and/or check-ups has a number of causes:

*Level of provision:*

Israeli-Arab settlements have relatively few dental clinics specially designed for children only, while getting to another settlement, sometimes a Jewish one, which does have such a clinic, can be expensive and time-consuming and impose some language and cultural difficulties.

*Costs:*

Despite the reform, even the remaining low out-of-pocket co-payment is enough to deter some families. Dentists interviewed said that they sometimes had to forgo asking for this payment.

*Cultural obstacles to accessing publicly-funded care:*

Some parents prefer or feel an obligation to get their dental care from a dentist working in the private sector, with whom they are friends or who belongs to their extended family (*hamula*), and will do so even at the price of missing the low-cost care opportunity available in the public sector. One interviewee recommended that HMOs sign up every dentist they can in every Israeli-Arab settlement in order to widen parents’ range of choice.

(b) The second and third stages

The aim of the interviews and the discussions with parents was to understand how they perceive the importance of maintaining dental health and the steps that need to be taken for this purpose and why they rarely take their children for check-ups. In addition, to reveal the factors and judgments influencing their choice of a dental health service for their children and identify obstacles to them taking up publicly-financed dental care. Analysing the data obtained from the face-to-face interviews and focus groups raised some insights relating to dental care use patterns of parents and children, and obstacles to the take-up of publicly-financed dental care.

### The parents’ own patterns of dental care use

Only a minority went for regular check-ups or to a hygienist. Many admitted to carelessness over dental care and check-ups, taking this as evidence that they could keep their teeth in good health without going to the dentist. Some even preferred self-treatment. A large proportion said that only severe pain would take them to a dentist. The explanations offered for this behaviour and these attitudes were the following:

*Fear:*

“Dentists frighten me. Only in an emergency situation will I go to one. I still prefer to suffer until I can no longer, when of course it costs much more and much more treatment has to be done. It’s fear of the injection, fear of the drill…” Many ascribed their present fears to bad personal experiences of dentists in childhood. They also told us of the bad dental care experiences their own children had had, which had left them, the parents, with a low opinion of dentists’ skills and their children very unwilling to return to the chair.

*Parents’ own living conditions as children*:

“As far as I can remember I never bothered about my teeth as a kid. There was no running water in the village, no tap in the house. You had to go outside and fill a bucket. We never bothered to go out into the cold and wind to clean our teeth.” One father said he was fifteen years old before he brushed his teeth.

### Patterns of care for children’s teeth

The overall picture that emerged was that parents’ and children’s personal preventive dental care at home were very similar. The majority of parents said that they enforced regular teeth brushing but several found this difficult. Some said they took practical steps to prevent too much eating of sweets or drinking of cola, others that this remained more a good intention than actual practice.

Few parents said they had started check-ups and treatment at an early age. Most of the parents who took their children to a dentist regularly said it started much later, usually when the first pain or other problem made itself felt.

“My boy is 9 years old and thank God I’ve never had to take him to a dentist, there’s never been any need.”

“Oh they all say that you should start check-ups early before they’re three years old but in practice they only do so when there’s pain.”

But even when a child claimed to be in pain, a trip to the dentist did not necessarily follow. One father said about his four-and-a-half-year- old:

“He often complains about pain and asks to be taken to the dentist but the truth is that I don’t, I don’t think it hurts as much as he says it does.”

Some cited their child’s fear of the drill or monetary concerns.

Ignorance about starting check-ups at the deciduous teeth stage was common:

"After all they’re going to fall out anyway” or ” The early teeth are stronger than the later ones” or “Up to age 5 they’re drinking milk so they’re getting good calcium and don’t need a dentist”.

### Obstacles to the take-up of publicly-financed dental care

#### Monetary obstacles

Many interviewees highlighted the high costs of dental treatments as a reason for postponing them until the pain can no longer be tolerated.

“Teeth care is not one of our priorities because it is so expensive …. so we put it off until the pain is too much to bear because every visit costs 300-400 NIS (New Israeli Shekels)”*.*

The financial barriers, which were mentioned regarding the treatment of the adults' teeth, also arose in the context of the treatment of the children's teeth. This is despite the possibility of receiving many of the treatments at a nominal cost in the public clinics and those that have agreements with HMOs. In this context, the deductibles for treatment in publicly funded clinics, as well as travel costs and other costs to the clinics, which are sometimes located in another settlement, were mentioned as barriers. This is especially difficult when there are several children in the family who need dental treatment.

“It mainly depends on the person's financial situation; for example, when I have 4-5 children, then it is difficult for me to go outside the village to get treatment. The same, before I go to HMO, I do my calculations, because there are fuel expenses, and payment of 27 NIS [the cost of the deductible for treatment], and not just for one child, but for four children, and when I want to go, then I take them all together, and sometimes they don't agree to let all four of them in, but only two or one”.

### Awareness and take-up of publicly-financed dental care

The majority of parents knew that publicly-financed dental care for children was available but not all knew the details, particularly the age limits. Some had heard of the new service from their HMO, others from the news media, others just happened to have heard about it. However, many did not know to what forms of treatment the new subsidy applied and were disappointed to learn of the gap between what they expected to be free care and the still existent demand for payment for elements of that care.

“They say that the price has dropped but every time I take my daughter to be examined, they say I still have to pay 150 NIS.”

“He did the examination and the X-rays and then said the X-rays were not included, so I had to pay 150 NIS for the X-rays and after that he says the treatment your son needs is also not included in the HMO service. So why did they tell me it was covered?”

Some parents said that when they found out that, in particular, orthodontic treatment and X-rays still had to be paid for they came to the conclusion that they might as well go to a private dentist. We received the clear impression that many still did not know what had to be paid for and how much and how much cheaper the HMO care was in the end than private care. Overall, there was disappointment in and distrust of the system and the information given to them.

### Satisfactions with the service

Although some parents expressed satisfaction with the new HMO service, many were dissatisfied with the quality, accessibility and availability of the service.

*Quality of provision*:

Some of the parents, who were not satisfied with their care, lost confidence in the quality of the service.

“I got the impression that the [dentists] were beginners and so I didn’t go back. I can’t entrust [my son] to just any one. I felt uncomfortable and then I didn't go back and preferred to go to a private doctor with my son. The main thing for me is to feel good and that the atmosphere will be comfortable so the child will receive a good care.” “It’s my feeling that you get what you pay for, if you pay little then what you get is worth little.”

*Access to and availability of provision:*

As noted above, not every HMO has a contracted clinic in every Arab town and village, so that some who want to take advantage of the new subsidized service are forced to travel to another settlement—which can entail difficulties:

“In my village there was only one dentist who had signed up with the HMO. If there are 4-5 dentists in the village the HMO should sign them all up so that we have some freedom of choice…”

“I used to have to travel to the [HMO] clinic in Nazareth but that became hard because of the journey from my village.”

“I started taking [my kids] to a dentist who signed a contract with my HMO but the waiting time started causing a problem. We had to wait a month or two months between appointments".

A mother who took her daughter to a clinic in a Jewish settlement said:

“They moved us from one dentist to another, and I remember that once we visited a dentist whom I don't know, we saw her only once. My daughter didn't feel comfortable there and started crying, so the doctor got annoyed. But it's not right to react like that especially since she is a doctor and my crying child is just a little girl.”

## Discussion

This research has explored the reasons for the low uptake by Israeli-Arab children of preventive dental care and of publicly-financed dental services in general.

A central explanatory factor in the low up take of preventive dental care is widespread lack of awareness of the importance of preventive care. The prevalent attitude is that one goes to the dentist only when there is a serious problem. Findings from several other studies have shown that parents' lack of knowledge and awareness about the importance of primary care and perceptions about it, create barriers to receiving preventive and primary care for children [16, 17]. The idea of early detection in order to prevent problems has not taken root. This is as equally true for the way Israeli-Arab parents care for their own teeth as for their children’s and it replicates the scant attention to preventive care by Israeli-Arabs in other aspects of healthcare [11, 18].

This carelessness reflects a lack of healthcare knowledge and awareness among Israeli-Arabs. Defining healthcare literacy as the ability to find, access and understand information about health and healthcare services and use that information to improve one’s own health, Levin, Zamir et al. [19] found it far lower among Israeli-Arabs than among Israeli Jews. The way to close this gap is to adapt services to the character of the Arab population, expand the availability, accessibility and the acceptability of those services, educate the public as to their importance, and energetically publicize them. School Dental Health Services have a positive effect on dental services utilization by children. Research conducted by Ashkenazi et al. [3] found that the letters the parents receive from the school dental health service after the dental exam advising them that the child has to see a dentist are effective and there is a high compliance. For this reason, too, it is important to ascertain that the School Dental Health Service is provided fully in the Arab sector.

Even when there is awareness of a service, this does not necessarily result in regular uptake. With respect to dentistry, one of the reasons for this is simply fear, fear of the drill and the pain it entails. This is not exclusive to Israeli-Arabs [20] but our interviews found it to be a common explanation not only of not making regular check-up visits but even of avoiding the treatment of actively painful problems. The parents attributed their fear to their bad dentistry experiences as children and said that their own children were still having similar experiences of dental care not adapted to children’s needs and sensitivities—mouths were still being forced open. These findings are consistent with other findings that emerged from a study conducted in Scotland [21] that examined the effect of parents' experience regarding their childhood dental care on their children's experience in the same matter and found that the sense of “uneasiness” that characterized parents' perceptions was provided for their children. The parents did remember their childhood experience and this was reflected in the delay of care for themselves and their children.

From both the interviewees’ responses and the preliminary interviews with service professionals it became clear that a contributor to this fear is the abovementioned habit of only going to the dentist when the problem has become insufferable, when of course the pain of the treatment is all the more severe. It is thus vital that parents be taught to take their children for regular check-ups starting at an early age, so that children learn to find the clinic unthreatening. For this to happen, a public education is needed and all dentists treating children must adapt both their methods and the physical environment of the clinic to children’s needs. Furthermore, oral healthcare providers should emphasize educational interventions and awareness raising actions directed to parents.

The study also reveals that there is awareness that public funding can be obtained, but there is a lack knowledge of which treatments are included in this arrangement. An important finding was that for low-income families, and particularly ones with many children, this is a real barrier. Over and above that, some Israeli-Arabs parents are not aware that the service is not totally free. This results in disappointment and frustration when faced with a demand to pay for treatment, which may lead to distrust in the system and even send these potential consumers back to non-HMO dentists. Thus, there is a need to HMOs to provide the public with the detailed information. School Dental Health Services letter to parents serves as important channel through which relevant health messages and information can be conveyed, as well. Another barrier is that the availability of public dental services provided by the HMOs should be improved in Israeli-Arab settlements. As mentioned, not all HMOs have contracted clinics in every Arab town or village. This means that some people may need to travel to another town to receive treatment, incurring travel costs and a larger time investment. This could be addressed by making sure that each one of the HMOs have contracts with at least one, and preferably more than one dental clinic in each town. Having contracts with a number of clinics will allow people to choose a clinic they prefer. HMO owned clinics are poli-clinics, not solo like the private clinics. The different set-up is sometimes strange and alarming to the children and their parents.

## Conclusions

From the findings of the present study and their discussion, it thus emerges that the factors which have deterred up take of the newly reformed dental service are a mixture of the subjective and objective. Subjectively, some parents have the impression that the service offered is not of a high quality, that it does not respect the personal and economic sensitivities of Israeli-Arab consumers. Objectively, barriers to the services still exist and it seems that the introduction of the reformed services has not sufficiently taken account of a long backlog of poor living conditions, fear of the drill, a low educational level which requires very transparent explication of such a new service, and the resistance of cultural traditions pressuring family members to take services from other extended family members [22].

## Recommendations

Steps should be taken to increase awareness in the Israeli-Arab community of the importance of preventive dental care—as early as the deciduous teeth stage—and of making regular preventive visits. This effort should include the importance of regular home brushing and healthy eating. Both parents and children should be targeted and schools, family doctors and dentists should be harnessed to this campaign.

Ways should also be found of lessening parents’ and children’s fear of the dental chair.

Israeli-Arabs should also be better informed of their rights to free and low-cost publicly-financed provided dental care for their children and, importantly, which treatments are free and which not, in order to avoid frustrations and resentment. In addition, Parents have to be informed precisely where the above services are available. The School Dental Health Services can incorporate a broader educational and explanatory initiative aimed at the parents too simultaneously with their children. This activity could encompass detailed information about the reform, the rights it grants, and how to utilize the service provided by the reform. It's important to highlight the role of family doctors in encouraging and guiding parents and their children in matters of oral and dental health. In Arab society in Israel, family doctors serve as a vital source of knowledge and information, extending beyond medical treatment. They offer guidance on health-related matters, disease prevention, and treatment options, acting as a trusted source of information within the community.

Lastly, the extent of the availability of these publicly-financed services should be examined. It's important to ensure that all the four HMOs set up an adequate service spread and adequate choice of dentists providing these services.

## Data Availability

Data and questionnaires are available from the corresponding author upon request.

## Abbreviations

NHIL: National Health Insurance Law
HMOs: Health Management Organizations
GPs: General Practitioners
MoH: Ministry of Health
DMFT: Decayed, Missing and Filled Teeth
